# Age-Related Changes in Matrix Metalloproteinase-9 Expression in Spinal Motor Neurons of Normal Mice

**DOI:** 10.7759/cureus.100305

**Published:** 2025-12-29

**Authors:** Jun Yamazaki, Takuto Hideyama, Sayaka Teramoto, Haruhisa Kato, Hitoshi Aizawa, Shin Kwak, Hiroo Terashi

**Affiliations:** 1 Neurology, Tokyo Medical University Hospital, Tokyo, JPN

**Keywords:** adenosine deaminase acting on rna 2 (adar2), amyotrophic lateral sclerosis (als), fast-fatigable motor neurons (ff mns), matrix metalloproteinase-9 (mmp-9), tar dna-binding protein 43 (tdp-43)

## Abstract

Amyotrophic lateral sclerosis (ALS) is a progressive neurodegenerative disorder involving the degeneration of upper motor neurons (UMNs) and lower motor neurons (LMNs). Although the cause of motor neuron (MN) degeneration in patients with ALS remains unknown, certain MN types (such as oculomotor neurons) and MNs within the Onuf (Onuf-Mannen) nucleus are preserved until the terminal stage. We previously generated mice with a selective knockout of adenosine deaminase acting on RNA 2 (ADAR2) in cholinergic neurons (*ADAR2^flox/flox^*/vesicular acetylcholine transporter (VAChT)-Cre.Fast; AR2). AR2 mice exhibit slow progressive loss of LMNs accompanied by TAR DNA-binding protein 43 (TDP-43) pathology against a background of insufficient editing at the GluA2 glutamine/arginine (Q/R) site due to ADAR2 deficiency. This model confirmed that insufficient editing at the GluA2 Q/R site, due to reduced ADAR2 activity, contributes to the pathogenesis of ALS. Furthermore, in AR2 mice, more frequent death of fast-fatigable motor neurons (FF MNs) was observed owing to differences in vulnerability under ADAR2-deficient conditions. Similar changes were observed during normal aging in the control mice. These findings suggest that investigating the characteristics of FF MNs may be useful for analyzing neuronal death in ALS. Recently, matrix metalloproteinase-9 (MMP-9), a marker of FF MNs, was reported to induce neurodegeneration. However, the distribution of MMP-9 in normal spinal MNs and its age-related changes remain unclear. Therefore, we investigated the MMP-9 expression patterns in normal mice at six and 12 months of age.

In the present study, the number of MNs in the anterior horn (AH) decreased with age, as did the number of MMP-9-positive MNs. Furthermore, as aging has been shown to induce the abnormal localization of TDP-43 in MMP-9-positive MNs, these MNs were considered vulnerable to degeneration.

These findings suggest that MMP-9 not only functions as a marker for FF MNs but may also act as a potentially useful marker for MNs prone to degeneration with TDP-43 pathology, or for early degeneration in both physiological aging and age-related diseases, including ALS. Future investigations of MMP-9 expression in patients with ALS and in ALS mouse models are considered useful for elucidating ALS pathogenesis.

## Introduction

Amyotrophic lateral sclerosis (ALS) is a progressive neurodegenerative disorder that affects the upper and lower motor neurons (MNs) [1]. In patients with ALS, MN damage leads to muscle weakness, skeletal muscle atrophy, and pathological reflexes. Approximately 90% of ALS cases are sporadic amyotrophic lateral sclerosis (SALS) and occur without genetic linkage, whereas approximately 20% of familial ALS (FALS) cases are caused by known mutations in the copper-zinc superoxide dismutase (*SOD1*) gene [2]. Molecular evidence for a common pathophysiology between SALS and FALS remains unknown. Although the etiology of MN degeneration in patients with ALS is unclear, certain MN types, including oculomotor neurons and MNs within Onuf’s (Onuf-Mannen’s) nucleus, are spared until the end-stage of the disease [3].

We previously generated mice (*ADAR2^flox/flox^*/vesicular acetylcholine transporters (VAChT)-Cre.Fast; AR2) with ADAR2 selectively knocked out in cholinergic neurons [4]. These AR2 mice displayed a slow, progressive loss of LMNs with TAR DNA-binding protein 43 (TDP-43) pathology in the context of insufficient editing at the GluA2 Q/R site due to ADAR2 deficiency [4], which has been observed only in MNs of sporadic ALS patients [5]. On the other hand, no reduction in RNA editing efficiency of the GluA2 Q/R site has been seen in MNs of rats transgenic for mutant SOD1 as well as patients with spinal and bulbar muscular atrophy [6]. Therefore, RNA editing abnormalities are considered a mechanism specific to sporadic ALS and not a phenomenon common to motor neuron diseases (MNDs). This model confirmed that inefficient editing at the GluA2 Q/R site resulting from reduced ADAR2 activity plays a role in the etiology of ALS.

Furthermore, death was more common among fast-fatigable (FF) MNs due to differences in vulnerability under ADAR2-deficient conditions in AR2 mice [7]. Similar changes occurred during normal aging in control mice [8]. The twitch force of fast motor units in electromyography is affected the earliest in patients with ALS [9], and FF motor units degenerate earlier than MNs that innervate slow muscles or those involved in eye movement and pelvic sphincter control in patients with ALS and aged subjects [10,11]. Moreover, FF MNs are selectively vulnerable to stress caused by axonal injury [12] and aging in wild-type mice [13,14]. Based on these results, investigating the properties of FF MNs could be useful for analyzing neuronal cell death in ALS.

Matrix metalloproteinase-9 (MMP-9), a positive marker of FF MNs [15], has been shown to trigger neurodegeneration [16,17] and increase MMP-9 expression in the spinal cord of Cu/Zn superoxide dismutase (SOD1^G93A^) mice [18]. However, its distribution in normal spinal MNs and age-related changes remains unknown. We hypothesized that MMP-9 expression in MNs is associated with aging and degeneration.

Accordingly, we investigated the MMP-9 expression patterns in normal mice at six and 12 months of age.

## Materials and methods

Ethics statement

This study was conducted in accordance with the Declaration of Helsinki, Tokyo Medical University Animal Experiment Guidelines, and National Institutes of Health guidelines. The experimental methods used in this study were approved by the Animal Handling Committee of Tokyo Medical University (study approval no. R6-007).

Mice

C57BL/6J mice (Oriental Yeast Co., Ltd., Tokyo, Japan) were used in this study. Animals were analyzed at six and 12 months old (n = 3 per group).

Antibodies

The following primary antibodies were used: rabbit anti-TDP-43 polyclonal antibody (Proteintech Group, Chicago, IL; dilution: 1:1,000), mouse anti-non-phosphorylated neurofilament H (SMI-32, Covance, Princeton, NJ; dilution: 1:1,000), and mouse anti-MMP-9 monoclonal antibody (Sigma-Aldrich, Saint Louis, MO; dilution: 1:5,000). The following species-appropriate secondary antibodies were used: Alexa Fluor 488 and 594 (Thermo Fisher Scientific, Waltham, MA; dilution: 1:1,000).

Immunohistochemistry

Under deep isoflurane anesthesia, the mice were transcardially perfused with 3% paraformaldehyde and 1% glutaraldehyde in phosphate-buffered saline (PBS). Spinal cords were removed and immersed sequentially in increasing concentrations of sucrose/PBS solution (final sucrose concentration: 30%) [7,8]. Immunohistochemical procedures were performed on 10-µm-thick sections cut using a cryostat (Model HM500 O; Epredia, Kalamazoo, MI) after rapid freezing with dry ice [7,8]. Sections mounted on slides were washed in PBS. Blocking solution was prepared (1% bovine serum albumin). The samples were then incubated with blocking solution for 1 h at 24 °C, and with primary antibodies (rabbit anti-TDP-43 polyclonal (dilution: 1:1,000), mouse anti-non-phosphorylated neurofilament H (dilution: 1:1,000), and mouse anti-MMP-9 monoclonal antibody (dilution: 1:5,000) for 1 h at 24 °C. After three washes with PBS for 5 min at 24 °C, the samples were incubated for 30 min with the secondary antibody in PBS containing the fluorescent nuclear probe 4′,6′-diamidino-2-phenylindole (DAPI, D1306; Molecular Probes, Eugene, OR; 1:200 dilution). After three washes, the samples were mounted using 15 μL of mounting solution (Vectashield Medium; Vector Laboratories Inc., Abcys, Paris, France) and an anti-fade reagent (Fluoromount/Plus K048; Diagnostic Biosystems, Pleasanton, CA, USA).

Morphological observations

The sections were observed and the data were analyzed using a fluorescence microscope (BIOREVO BZX-800; Keyence, Osaka, Japan).

**Figure 1:**
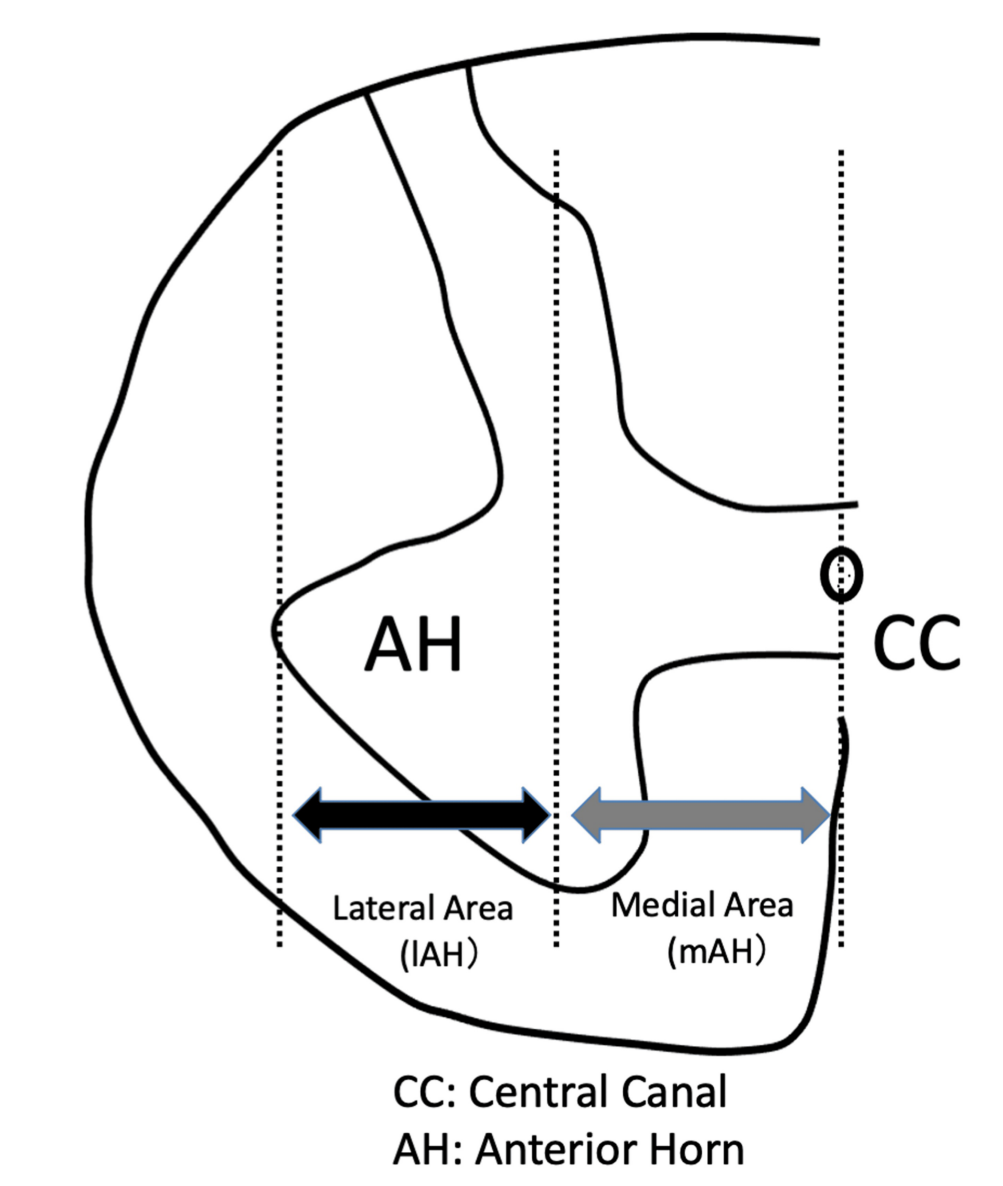
Definition of the lateral area of the anterior horn (lAH) and medial area of the anterior horn (mAH) within the anterior horn (AH) of the spinal cord. The lateral and medial areas are separated at the midpoint between the central canal of the spinal cord and outer edge of the AH, adapted from a previous study [7] with permission (Copyright 2025 by "Elsevier;" No. 615407138996). The AH of the spinal cord is part of the spinal gray matter situated ventral to the central canal.

The medial anterior horn (mAH) and lateral anterior horn (lAH) were separated at the midpoint between the central canal and the outer edge of the anterior horn (AH) (Figure 1) [7]. MNs were counted and measured in cross-sections with the maximum diameter, which contained the nucleus; methods for measuring neuron diameter were used from previous reports [7,8].

**Figure 2:**
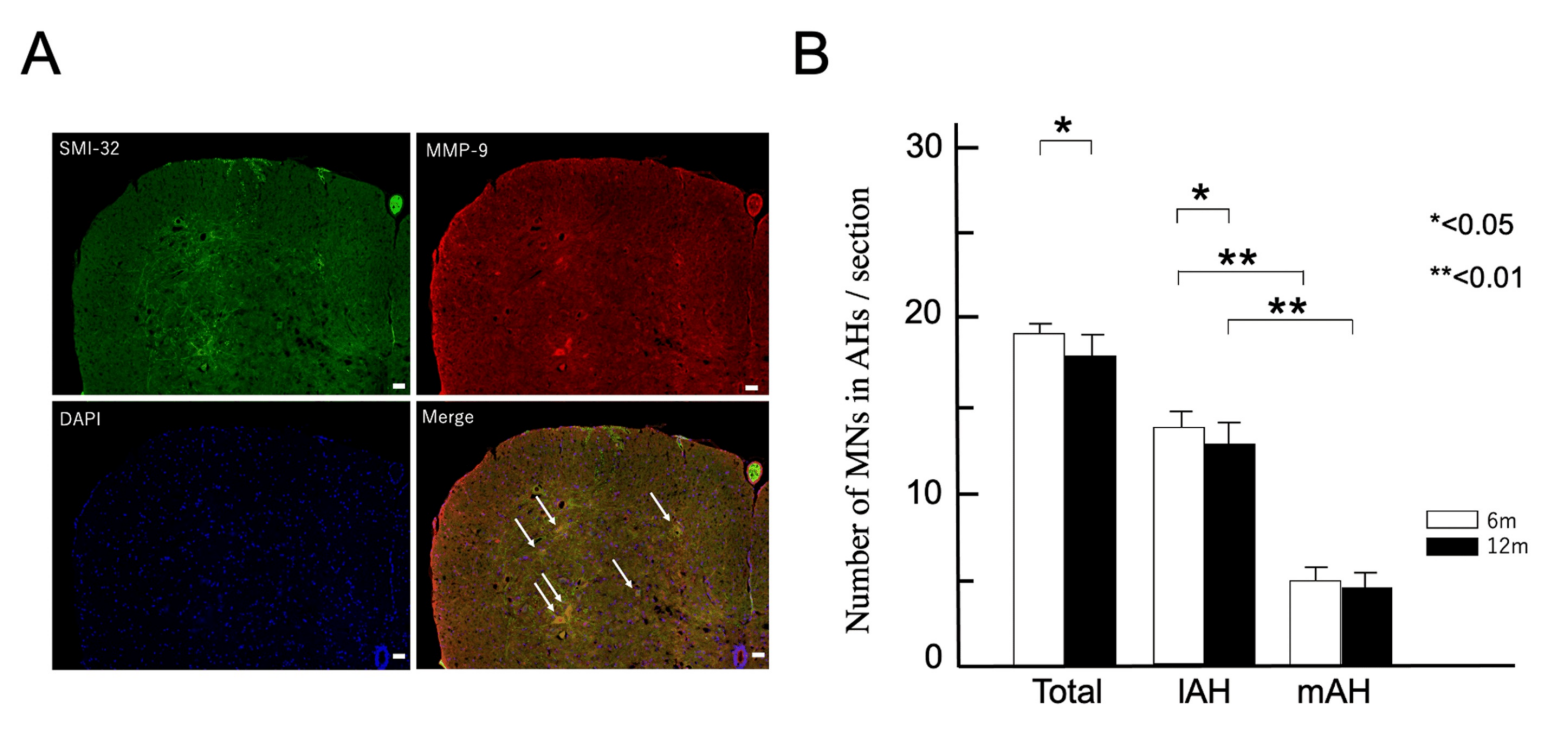
Decrease in motor neurons (MNs) in the lAH. (A) Immunostaining for non-phosphorylated neurofilament H (SMI-32; green), matrix metalloproteinase-9 (MMP-9; red), and the fluorescent nuclear probe 4′,6′-diamidino-2-phenylindole (DAPI; blue) in the AH of the spinal cord of 12-month-old mice. White arrows indicate representative SMI-32- and MMP-9-positive MNs. Scale bar: 10 μm. (B) Numbers (mean ± standard error of the mean) are shown for MNs. The number of MNs is significantly lower in the AHs and lAH of the 12-month-old mice (p < 0.05). The number of MNs in the mAH is significantly lower in six- and 12-month-old mice (**p < 0.01). Columns show six-month-old (6m; white) and 12-month-old (12m; white) mice. AH: Anterior horn; lAH: Lateral anterior horn

Sections of the lumbar spinal cord (L5) were double-stained for SMI-32 and MMP-9 using immunofluorescence (Figure 2A). MNs were stained with SMI-32, and those with a typical morphology and large soma (diameters > 20 μm) in the mAH and lAH of the lumbar spinal cord (L5) of 6 and 12-month-old wild-type (WT) mice (n = 3) were counted.

Previously, we examined the sections of the fifth cervical (C5) and fifth lumbar (L5) spinal cord segments which were sequentially immunostained with SMI-32 using the HRP-DAB system in AR2 mice at 12 months of age [4]. As a result, both the number of C5 MNs and L5 MNs showed a reduction in AR2 mice compared to age-matched normal control mice. This phenomenon was common to both the cervical and lumbar spinal cords in AR2 mice. Furthermore, since the number of MNs is greater in the lumbar AH than in the cervical AH, which makes changes easier to detect. Hence, we analyzed the number of MNs in the lumbar AH (L5). Sections of the lumbar spinal cord (L5) were sequentially immunostained for MMP-9 and TDP-43, using an immunofluorescence system. The number of MNs in which TDP-43 was detected in the cytoplasm was counted in 10 L5 sections from each mouse. We classified the TDP-43 staining patterns of MMP-9-positive MNs into two types. In Type 1, TDP-43 was present in the nucleus and absent in the cytoplasm. This is the normal pattern of staining in MNs and is most common in six-month-old mice. In Type 2, TDP-43 was present in both the nucleus and cytoplasm and was observed only in 12-month-old mice. Types 1 and 2 were counted by two researchers who were unaware of the age of the mice.

Statistical analyses

Differences and correlations between the two groups were evaluated using the Mann-Whitney U test with IBM SPSS Statistics for Windows, Version 29 (Released 2023; IBM Corp., Armonk, New York, United States). Data are shown as the mean ± standard error of the mean (SEM). Differences were considered statistically significant at p < 0.01 and highly significant at p < 0.001.

## Results

Effect of age on the number of MNs in the lAH

In both six- and 12-month-old WT mice, large-diameter MNs (≥20 μm) were more in the lAH (6m mAH: 5.4 ± 0.11; lAH: 13.4 ± 0.16, p < 0.001, 12m mAH: 5.2 ± 0.20; lAH: 12.4 ± 0.17, p < 0.001, Figure 2B). Comparing six- and 12-month-old WT mice, the number of SMI-32-positive MNs decreased significantly (6m: 18.9 ± 0.50; 12m: 17.6 ± 0.36, p < 0.05, Figure 2B), particularly in the lAH (6m: 13.4 ± 0.16; 12m: 12.4 ± 0.17, p < 0.05, Figure 2B) while the number in the mAH remained unchanged (6m: 5.4 ± 0.11; 12m 5.2 ± 0.20, p > 0.05, Figure 2B). Thus, large-diameter MNs in the lAH account for the age-related degeneration of MNs.

Predominant localization of MMP-9-positive MNs in the lAH

**Figure 3:**
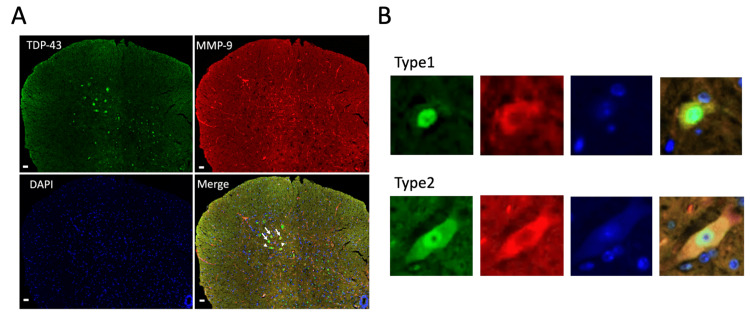
MMP-9-positive MNs and type classification of MNs in the lAH. (A) Representative confocal images of the mouse AH. Immunostaining for TAR DNA-binding protein 43 (TDP-43; green), MMP-9 (red), and the fluorescent nuclear probe DAPI (blue) in the AH of the spinal cord of 12-month-old mice. Scale bar: 10 μm. (B) TDP-43 staining patterns of MMP-9-positive MNs into two types. Type 1 MNs: TDP-43 staining occurs only in the nucleus. In type 2 MNs, TDP-43 staining is observed strongly in the nucleus and faintly in the cytoplasm. White arrows indicate representative Type 1 MNs and white arrowheads indicate representative Type 2 MNs in (A). MN: Motor neuron; MMP-9: Matrix metalloproteinase-9; AH: Anterior horn; lAH: Lateral anterior horn

**Figure 4:**
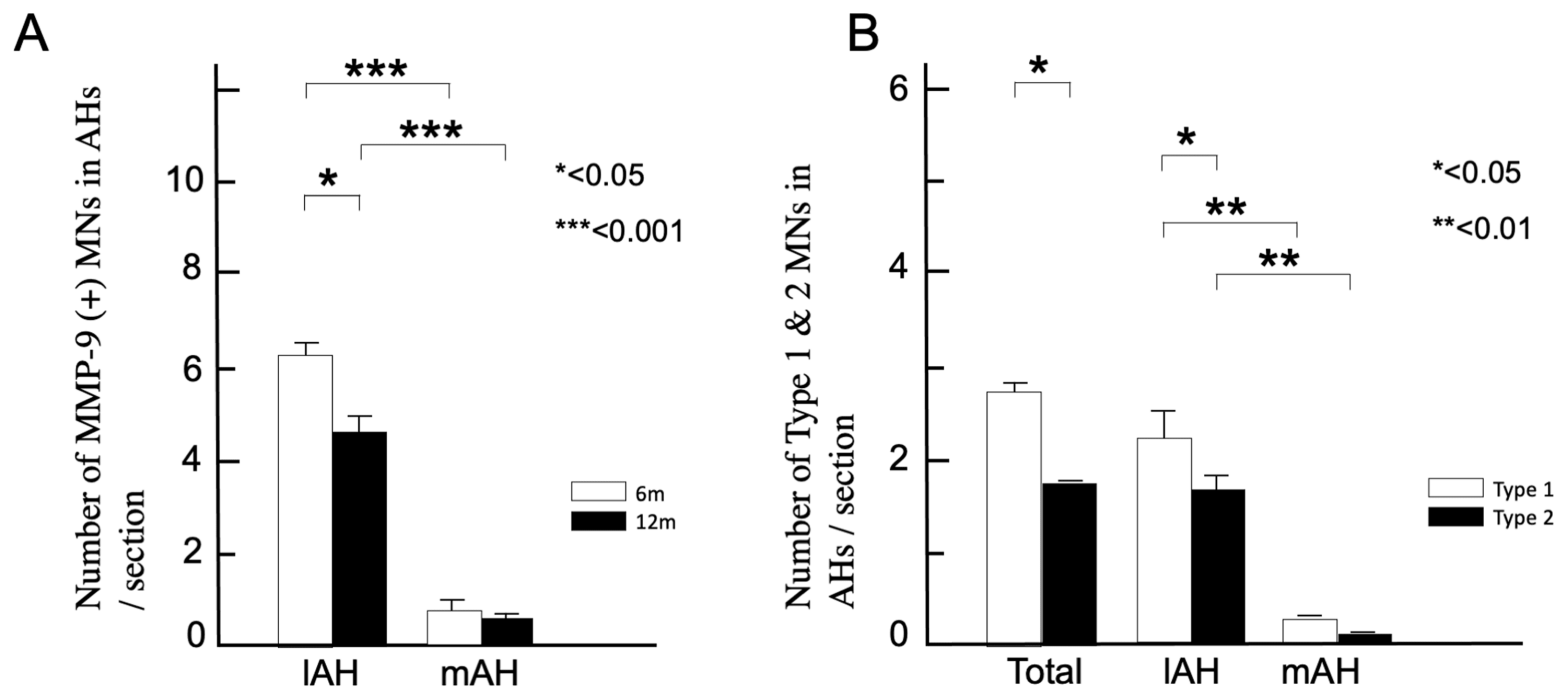
With aging, MMP-9-positive MNs in the lAH with TDP-43 mislocalization are significantly reduced. (A) In 12-month-old WT mice, MMP-9 positive MNs are significantly reduced in the lAH (6 months: 6.1 ± 0.17; 12 months lAH: 4.6 ± 0.15; *p < 0.05). The number of MMP-9 positive MNs in the mAH is significantly lower in six- and 12-month-old WT mice (***p < 0.001). Columns show six-month-old (6m; white) and 12-month-old (12m; white) mice. (B) Among the total number of MNs in the AH, Type 1 MNs are significantly more numerous than Type 2 MNs (*p < 0.05) and are significantly more frequently observed in the lAH than that in the mAH (**p < 0.01). Type 2 MNs are significantly more frequently observed in lAH (lAH: 1.6 ± 0.06; mAH: 0.63 ± 0.06; * *p* < 0.01). MN: Motor neuron; MMP-9: Matrix metalloproteinase-9; AH: Anterior horn; lAH: Lateral anterior horn; mAH: Medial anterior horn

MMP-9 and TDP-43 staining of the AH is shown in Figure 3A. In six- and 12-month-old WT mice, MMP-9-positive MNs were significantly more numerous in the lAH than in the mAH (6m mAH: 0.87 ± 0.15; lAH: 6.1 ± 0.17; p < 0.001; 12m mAH: 0.63 ± 0.06; lAH: 4.6 ± 0.15; p < 0.001, Figure 4A). Thus, MMP-9-positive MNs were predominantly localized in the lAH.

Effect of aging on MMP-9-positive MNs in the lAH

The proportion of MMP-9-positive MNs in the lAH was significantly reduced in 12-month-old mice (31.7 ± 1.0%) than in those aged six months (39.0 ± 2.0%), while that in the mAH of 12-month-old mice 12 months (12.2 ± 1.2%) was comparable to those aged six months (16.0 ± 3.1%). These results indicate that MMP-9 positive MNs were significantly reduced in the lAH (6m: 6.1 ± 0.17; 12m: 4.6 ± 0.15; p < 0.05, Figure 4A).

Effect of aging on MMP-9-positive MNs with abnormal TDP-43 localization in the lAH

MMP-9-negative MNs exhibited a normal pattern, in which TDP-43 remained confined to the nucleus in six- and 12-month-old WT mice. Since TDP-43 was specifically localized in the nucleus in normal MNs (Type 1 in Figure 3) but localized in both the nucleus and the cytoplasm with age-dependent degenerative process (Type 2 in Figure 3), we next investigated whether MMP-9-positive MNs in the lAH are specifically vulnerable to age-dependent degeneration. In 12-month-old WT mice, a significant proportion of the MMP-9-positive MNs exhibited a Type 2 pattern, a pattern seen in the degenerating MNs (lAH: 41.6 ± 0.06; mAH: 0.63 ± 0.06; p < 0.05, Figure 4B), suggesting the specific vulnerability of MMP-9-positive MNs in the lAH to age-related degeneration.

## Discussion

In this study, we demonstrated that MMP-9 expression in the mouse spinal cord was strongly associated with the MNs in the lateral AH, and that these MMP-9-positive MNs were prone to age-related degeneration with TDP-43 mislocalization. Since MMP-9-positive MNs can be used as markers for FF MNs [15,18,19] and FF MNs have been known to be vulnerable to age-related degeneration [13,14], here we have provided additional evidence that MMP-9-positive MNs in the lAH, supposed to be FF MNs, are vulnerable to aging.

MMP-9 is a protease that acts extracellularly and extrasynaptically and is significantly activated in response to environmental damage. It plays a crucial role in excitatory synaptic plasticity, and its abnormal expression has been implicated in the pathogenesis of psychiatric disorders [20]. Recently, MMP-9 has been reported to be linked to aging and degenerative diseases, such as Alzheimer’s disease [21] and Werner syndrome [22]. The crucial function of MMP-9 associated with degenerative and age-related changes is to cause barrier leakage by compromising the integrity of the blood-brain barrier, resulting in the infiltration of inflammatory molecules (such as inflammatory mediators), extravasation, and invasion of immune cells into the brain [23]. These functions are also associated with degenerative and age-related changes. Consequently, targeting MMP-9, either by inhibiting its activity or preventing its expression, has been proposed as a potential therapeutic strategy for controlling age-related inflammation and neurodegenerative processes [20,24].

Based on the above lines of evidence, this study suggests that MMP-9 may serve not only as a marker for FF MNs, but also potentially as a marker for MNs prone to degeneration with TDP-43 pathology or for early degeneration in both physiological aging and age-related diseases, including ALS.

Aging is a risk factor for many neurodegenerative diseases, including ALS. Most patients with ALS develop the initial symptoms in middle age or later, and ALS incidence increases with age [25,26]. One reason may be an age-related decline in ADAR2 activity in lAH MNs [8]. ADAR2 activity has been reported to decline with age in lAH MNs, leading to decreased editing efficiencies and TDP-43 pathology [8].

These findings indicate that aging induces abnormal TDP-43 localization in MMP-9-positive MNs, which are considered FF MNs. Therefore, MMP-9-positive MNs may be prone to degeneration.

This study has some limitations. The sample size is small, and the analysis was limited to ages six and 12 months. Therefore, further investigation is needed to determine when Type 2 begins to be observed after the six-month transition. Additionally, regarding the frequency of Type 2 and the abnormal localization of TDP-43, we would like to investigate these aspects in the future if older cases beyond 12 months of age become available. The extent to which the role of MMP-9 can be generalized beyond ALS to other MND remains a limitation that requires further discussion. We will investigate the distribution of MMP-9 in AR2 mice to determine whether our findings support disease-specific mechanisms or indicate more universal pathogenic pathways.

## Conclusions

Further investigations of MMP-9 expression in patients with ALS and ALS mouse models are considered useful for elucidating ALS pathogenesis. Therefore, we plan to use our AR2 mice to investigate the relationship between ADAR2 and MMP-9 in MNs, as well as the expression of MMP-9 in degenerating MNs.
